# The American Medical Association

**Published:** 1895-02

**Authors:** 


### The American Medical Association.

With the close of the session at San Francisco the following officers were elected for the ensuing year: President, Doctor Donald Maclean, of Michigan; Vice-President, T. C. Loveling, of Ohio; Treasurer, Doctor Newman, of Illinois; Secretary, William B. Atkinson, of Pennsylvania; Assistant Secretary, H. B. Ellis, of California; Librarian, G. E. Webster, of Illinois; Editor of the Association *Journal*, J. B. Hamilton, of Illinois.—*The Medical Age.*

